# Surgery of ascending aorta with complex procedures for aortic dissection through upper mini-sternotomy versus conventional sternotomy

**DOI:** 10.1186/s13019-020-01095-1

**Published:** 2020-04-07

**Authors:** Yang Wu, Wei Jiang, Dong Li, Lei Chen, Weihua Ye, Chonglei Ren, Cangsong Xiao

**Affiliations:** Department of Cardiovascular Surgery, PLA General Hospital, 28 Fuxing Road, Beijing, China

**Keywords:** Aortic dissection, Upper mini-sternotomy, Standard propensity score matching

## Abstract

**Background:**

Use of minimally invasive approaches for isolated aortic valve or ascending aorta surgery is increasing. However, total arch replacement or aortic root repair through a minimally invasive incision is rare. This study was performed to report our initial experience with surgery of the ascending aorta with complex procedures through an upper mini-sternotomy approach.

**Methods:**

We retrospectively analyzed 80 patients who underwent ascending aorta replacement combined with complex procedures including hemi-arch, total arch, and aortic root surgeries from September 2010 to May 2018. Using standard propensity score-matching analysis, 36 patients were matched and divided into 2 groups: the upper mini-sternotomy group (*n* = 18) and the median sternotomy group (n = 18). The preoperative assessment revealed no statistically significant differences between the two groups.

**Results:**

Hospital mortality occurred in one patient (2.8%). The mini-sternotomy group showed a longer cross-clamping time (160 ± 38 vs. 135 ± 36 min, *p* = 0.048) due to higher rate of valve-sparing aortic root replacement and total arch repair. The cardiopulmonary bypass time in mini-sternotomy group was shorter than that of full sternotomy group (209 ± 47 min vs 218 ± 62 min, *p* = 0.595) but fell short of significance. There was no significant difference in lower body hypothermia circulatory arrest time between the two groups (40 ± 10 min vs 48 ± 20 min, *p* = 0.139). The upper mini-sternotomy group displayed a shorter ventilation time (22 vs. 45 h, *p* = 0.014), intensive care unit stay (4.6 ± 2.7 vs. 7.9 ± 3.7 days, *p* = 0.005), and hospital stay (8.2 ± 3.8 vs. 21.4 ± 11.9 days, *p* = 0.001). The upper mini-sternotomy group showed a lower postoperative red blood cell transfusion volume (4.6 ± 3.3 vs. 6.7 ± 5.7 units, *p* = 0.042) and postoperative drainage volume (764 ± 549 vs. 1255 ± 745 ml, *p* = 0.034). The rates of dialysis for newly occurring renal failure, neurological complications, and re-exploration were similar between the two groups (*p* = 1.000).

**Conclusion:**

The upper mini-sternotomy approach is safe and beneficial in ascending aorta surgery with complex procedures for aortic dissection, including total arch replacement and aortic root repair.

## Introduction

Surgical developments have led to faster recovery with a shorter hospital stay, enhanced thoracic stability, reduced pain, and superior cosmetic results. Consequently, the use of a minimally invasive approach through an upper mini-sternotomy for isolated aortic valve surgery or combined aortic root and ascending aorta surgery is finding wide consensus and spreading further among cardiac surgery centers worldwide [1–4]. However, ascending aorta surgery with or without combined aortic arch surgery, especially total arch surgery, is not yet widely performed through a minimally invasive surgical incision. A full median sternotomy remains the standard approach for complex aortic surgery to ensure adequate exposure and safety [5]. However, some experts attempt to perform minimally invasive procedures in aortic arch surgery [6, 7]. The present study was performed to demonstrate that complex aortic surgery including total arch surgery and aortic root repair via a partial upper sternotomy is viable, safe, and equivalent to the standard procedure in terms of both safety and the risk of major adverse cardiac and cerebrovascular events.

## Materials and methods

### Patients

From September 2010 to May 2018, 80 open heart operative replacements of the thoracic aorta for treatment of aortic dissection were performed by one surgeon in Chinese PLA General Hospital. Patients with isolated non-dissected aortic aneurysms were excluded. Among the 80 patients, 21 underwent operations through an upper mini-sternotomy and 59 underwent operations through a full sternotomy. Considering that the complexity of the clinical data may impact the results, we used standard propensity score matching to create a highly comparable control group. Thus, 18 patients in each group (upper mini-sternotomy group and median sternotomy group) were matched for the statistical analysis. In the upper mini-sternotomy group, 12 of 18 patients underwent emergency ascending aorta replacement combined with hemi-arch or total arch replacement and/or aortic root repair; 13 of 18 patients underwent concomitant total arch replacement, 3 underwent hemi-arch replacement, and 2 underwent ascending aorta replacement only; 9 of 18 patients underwent concomitant aortic root repair, 4 underwent valve-sparing aortic root replacement (David procedure using re-implantation technique), 1 underwent Florida sleeve root repair, 4 underwent isolated aortic valvuloplasty, and 1 underwent wrapping of the aortic root with an artificial vascular prosthesis. In the matched group, 14 of 18 patients underwent emergency surgery for the ascending aorta combined with hemi-arch or total arch and/or aortic root replacement; 11 of 18 patients underwent concomitant total arch replacement, 6 of 18 underwent hemi-arch replacement, and 10 underwent concomitant aortic root repair (Bentall procedure in 4, aortic valvuloplasty in 5, and valvuloplasty with coronary artery ostia grafting in 1). The indication for aortic repair was based on the standard guidelines and was at the discretion of the multidisciplinary team. The baseline characteristics of the patients of each group are shown in Table 1. Overall, the individual preoperative risk factors, including the EuroSCORE, were similar between the two groups.

**Table 1:** Patients’ baseline characteristics

	Upper ministernotomy (***n*** = 18)^**a**^	Median sternotomy (***n*** = 18)^**a**^	***P*** value
**Age**	**45.6 ± 13.6**	**49.6 ± 11.5**	**0.340**
**Male**	**12 (0.67)**	**14 (0.78)**	**0.457**
**BMI**	**26.39 ± 4.84**	**25.44 ± 2.43**	**0.465**
**Hypertension**	**13 (0.72)**	**13 (0.72)**	**1**
**Marfan’s syndrome**	**1 (0.06)**	**1 (0.06)**	**1**
**Diabetes mellitus**	**0**	**0**	**1**
**COPD**	**0**	**0**	**1**
**PAD**	**3 (0.17)**	**3 (0.17)**	**1**
**renal insufficiency**	**1 (0.06)**	**2 (0.11)**	**1**
**Dialysis**	**0**	**0**	**1**
**Stroke**	**1 (0.06)**	**1 (0.06)**	**1**
**CAD**	**2 (0.11)**	**1 (0.06)**	**1**
**AI**			**0.340**
None	**10 (0.56)**	**8 (0.44)**	
Mild	**2 (0.11)**	**4 (0.22)**	
Moderate to severe	**6 (0.33)**	**6 (0.33)**	
**LVEF (%)**	**66 ± 9**	**65 ± 7**	**0.623**
**EuroSCORE**	**5.33 ± 1.41**	**5.40 ± 2.10**	**0.914**

*BMI* body mass index, *COPD* chronic obstructive pulmonary disease, *PAD* peripheral artery disease, *CAD* coronary artery disease, *AI* aortic insufficiency, *LVEF* left ventricular ejection fraction

^a^Continuous data are presented as mean ± standard deviation, categoric data as number (%)

### Surgical technique

**Fig. 1:**
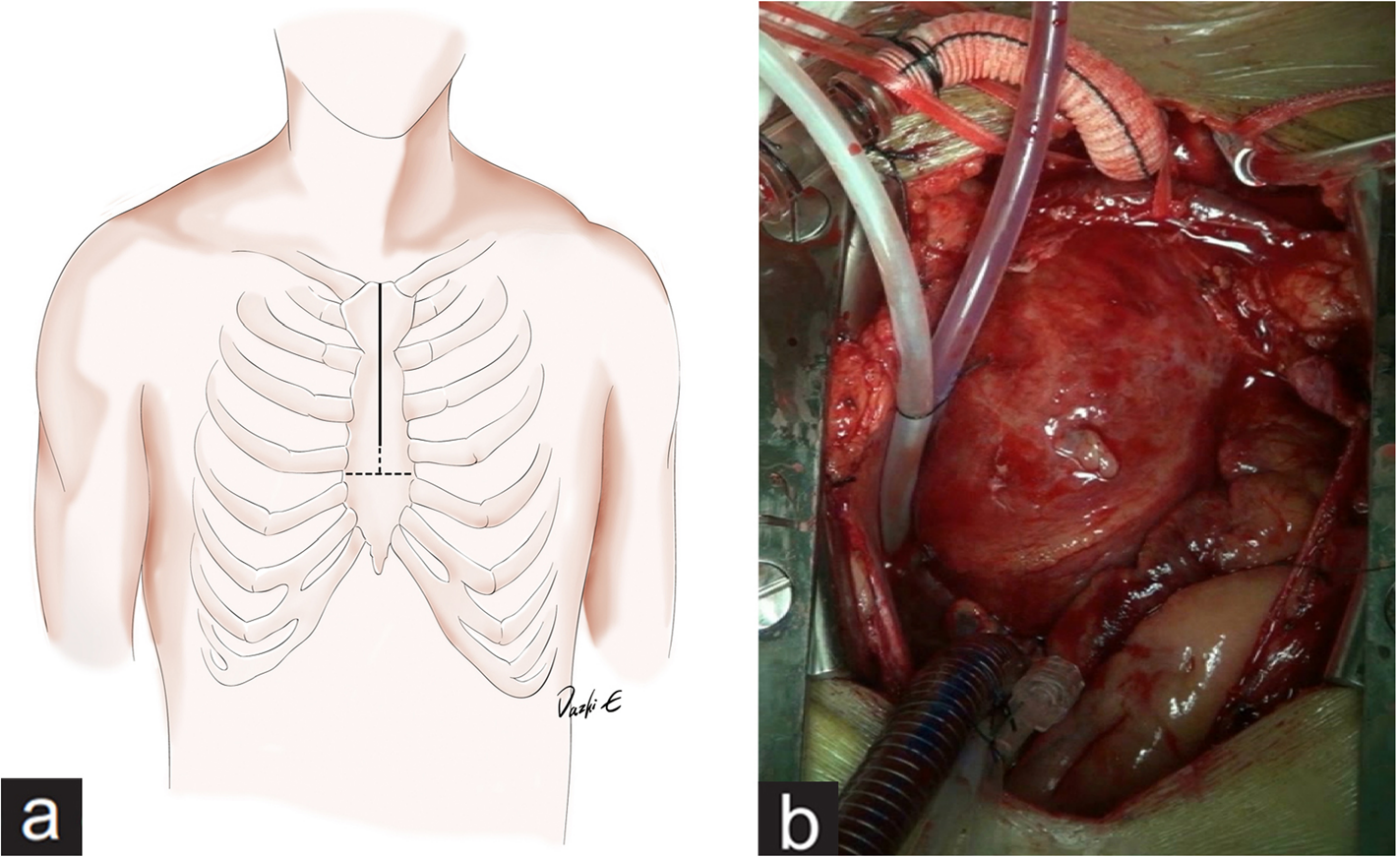
(**a**) upper inverted-T mini-sternotomy (**b**) CPB was established

Our upper mini-sternotomy approach is similar to those described in previous reports. The patient was maintained in the supine position, and a single-lumen endotracheal tube was used for ventilation. The internal jugular vein was catheterized to monitor the central venous pressure and pulmonary artery pressure and provide an infusion pathway. A urethral catheter was routinely placed. After placing the patient under general anesthesia, we performed a 6- to 8-cm median skin incision and upper inverted-T mini-sternotomy to the level of the third or fourth intercostal space depending on preoperative computed tomography scan. A conventional sternal retractor was used to spread the sternum and expose the substernal tissue (Fig. 1a). The innominate vein and three branches of the aortic arch were then dissected separately. Once the pericardium was opened, the ascending aorta was exposed along its entire length. The strategy of cardiopulmonary circulation setup depended on the aortic anatomy and lesion characteristics. We generally selected cannulation of the innominate artery or axillary artery for perfusion and the right atrium for drainage. The left heart venting was inserted through the upper right pulmonary vein (Fig. 1b). Myocardial protection was routinely implemented by anterograde infusion of cold custodial Histidine-tryptophan-ketoglutarate (HTK, Bretschneider’s solution, Custodiol) cardioplegic solution directly through the coronary ostia. Carbon dioxide in the surgical field was used to reduce the risk of air embolism. Surgery of complex aortic lesions with or without concomitant aortic valve and root surgery was performed as necessary. Lower-body circulatory arrest was used in some patients who required total arch replacement and elephant trunk implantation. We preferred an “arch-first” strategy for arch replacement surgery. In these cases, we first accomplished reconstruction of the branches of the aortic arch [left subclavian artery (LSCA), left carotid artery (LCA), and innominate artery (IA)] using a four-branched graft with or without cardiopulmonary bypass (CPB) to recover complete cerebral blood perfusion. When the bladder temperature had cooled to about 25 °C, the lower body circulation was arrested and upper body (including cerebral) was perfused via the preferentially reconstructed supra-aortic branches, which we named it as “complete antegrade cerebral perfusion”. We then performed frozen elephant trunk implantation in the descending aorta and arch reconstruction if necessary. In the rewarming period, proximal surgery such as root or ascending aorta reconstruction was performed. Deairing was routinely performed before declamping the aorta. A ventricular pacing wire was placed before declamping to allow for better exposure of the right ventricular wall via the minimal surgical access. Two straight 26-Fr thoracic catheters were inserted through subcostal incisions and connected to a water seal multichamber thoracic drainage device. The sternum was reconstructed with three single steel wires and breadthwise suture placement in a figure-eight pattern.

### Statistical analysis

We used standard propensity score matching (1:1 ratio) based on sex, age, body mass index, complications, ejection fraction, EuroSCORE and type of surgery to divide the patients into two groups. The upper mini-sternotomy group was defined as the observation group, and the median sternotomy group was matched as the control group. Continuous variables are expressed as mean ± standard deviation (or median when the distribution of variables is not normally distributed) and were compared using Student’s t-test or Wilcoxon rank sum test (for nonnormally distributed variables). Categorical variables are expressed as percentages and were compared using χ^2^ test or Fisher exact test when the number of patients in each cell was smaller than five. All *p*-values of < 0.05 were considered statistically significant. The statistical analyses were performed using SPSS 22.0(Statistical Package for Social Sciences, Microsoft).

## Results

The intraoperative variables are listed in Table 2. The CPB time was similar between the two groups (209 ± 47 min vs 218 ± 62 min, *p* = 0.595), but the aortic cross-clamping time was longer in the upper mini-sternotomy group than that of full sternotomy group (160 ± 38 vs. 135 ± 36 min, *p* = 0.048) due to higher rate of valve-sparing aortic root replacement and total arch repair. The hypothermic circulatory arrest (HCA) time was similar between the two groups(40 ± 10 min vs 48 ± 20 min, *p* = 0.139), indicating that no more time was spent on descending aorta reconstruction in the upper mini-sternotomy group than in the control group.

**Table 2:** Intraoperative variables

	Upper ministernotomy(***n*** = 18)^**a**^	Median sternotomy(n = 18)^**a**^	P value
**Catogeries of surgery**			**0.095**
ASA + hemi-arch	**1 (0.06)**	**6 (0.33)**	
ASA + total arch	**13 (0.72)**	**8 (0.44)**	
root+ASA	**2 (0.11)**	**1 (0.06)**	
root+ASA + hemi-arch	**0**	**2 (0.11)**	
root+ASA + total arch	**2 (0.11)**	**1 (0.06)**	
**CBP time (min)**	**209 ± 47**	**218 ± 62**	**0.595**
**Crossclamp time (min)**	**160 ± 38**	**135 ± 36**	**0.048**
**HCA time (min)**	**40 ± 10**	**48 ± 20**	**0.139**

*ASA* ascending aorta, *CPB* cardiopulmonary bypass, *HCA* hypothermic circulatory arrest

^a^Continuous data are presented as mean ± standard deviation, categoric data as number (%)

The postoperative morbidities are listed in Table 3. Of all 36 patients, only 1 died in the observation group, and no significant difference was found in comparison with the control group (*p* = 1.000). The red blood cell transfusion volume (4.6 ± 3.3 vs. 6.7 ± 5.7 units, *p* = 0.042), postoperative drainage volume (764 ± 549 vs. 1255 ± 745 ml, *p* = 0.034), ventilation time (22 vs. 45 min, *p* = 0.014), intensive care unit (ICU) stay (4.6 ± 2.7 vs. 7.9 ± 3.7 days, *p* = 0.005), and hospital stay (8.2 ± 3.8 vs. 21.4 ± 11.9 days, *p* = 0.001) were all significantly lower in the upper mini-sternotomy group. The rate of subxiphoid drainage for late cardiac tamponade was significantly higher in the observation group than control group (33.3% vs. 5.6%, *p* = 0.004). The rate of dialysis for new occurrence of renal failure, neurological complications, and re-exploration was similar between the two groups (*p* = 1.000).

**Table 3:** Postoperative outcomes

	Upper ministernotomy (n = 18)^**a**^	Median sternotomy (n = 18)^**a**^	P value
**RBC transfusion(U)**	**4.6 ± 3.3**	**6.7 ± 5.7**	**0.042**
**Drainage volume (ml)**	**764 ± 549**	**1255 ± 745**	**0.034**
**Pericardial puncture for late tamponade**	**7 (0.33)**	**1 (0.06)**	**0.004**
**Ventilation (h)**	**22 (13,88)**	**45 (25,122)**	**0.014**
**ICU stay(d)**	**4.6 ± 2.7**	**7.9 ± 3.7**	**0.005**
**Hospital stay(d)**	**8.2 ± 3.8**	**21.4 ± 11.9**	**0.001**
**New occurred renal failure (dialysis)**	**2 (0.11)**	**1 (0.06)**	**1**
**Neurological complications**	**2 (0.11)**	**3 (0.17)**	**1**
**Re-exploration**	**1 (0.06)**	**1 (0.06)**	**1**
**Death**	**1 (0.06)**	**0**	**1**

*RBC* red blood cell, *ICU* intensive care unit

^a^Continuous data are presented as mean ± standard deviation or median; categoric data as number (%)

## Discussion

Surgery of the ascending aorta with or without combined procedures, such as total arch replacement and aortic root reconstruction, has been traditionally performed through a median full sternotomy because this procedure can provide good exposure for deep surgical operations, especially when descending aorta management or aortic root procedures are required. Surgeons have often considered that the performance of a distal anastomosis in a deep position is technically challenging and that approaching the aorta beyond the reflection point of the arch via a median sternotomy is generally difficult [6]. Nowadays, however, the development of minimally invasive techniques has allowed increasingly improved results of aortic surgery, not only for isolated heart valve disease but also for ascending aorta repair, the Bentall procedure, and hemi-arch replacement. In addition to its cosmetic benefits, the minimally invasive approach for valvular surgery has been shown to improve postoperative outcomes; reduce surgical trauma, the need for ventilation, the ICU stay, and the need for blood transfusion; and decrease the incidence of respiratory failure compared with full sternotomy [1, 2].

Different minimally invasive approaches have been reported for cardiac surgery [7, 8]. Widespread consensus has been reached regarding the efficacy of upper J mini-sternotomy for the treatment of aortic disease [2, 9, 10]. The indication for surgery, initially restricted only to selected patients, is now extended to those undergoing more complex surgeries, including surgeries involving the aortic root and ascending aorta as well as redo operations. The mini-sternotomy approach has been shown to be beneficial for respiratory function recovery, earlier extubation, and a shorter ICU and hospital stay [11]. In 2007, Tabata et al. [1] compared 79 patients undergoing mini-sternotomy with a cohort of patients undergoing surgery through full sternotomy. Their study was remarkable with respect to the variety of different procedures completed using the mini-sternotomy approach. In 2001, Svensson et al. [12] evaluated 54 patients undergoing minimally invasive valve surgery; of these, 36 patients underwent ascending aorta replacement, of whom 18 underwent aortic arch repair through a mini-sternotomy. In 2017, Goebel et al. [13] reported the outcomes of 21 patients who underwent non-emergency total aortic arch surgery through an upper mini-sternotomy. These patients’ results were equivalent to those of patients who underwent the standard procedure in terms of both safety and the risk of major adverse cardiac and cerebrovascular events.

In the present study, we attempted to perform surgery of the ascending aorta with or without complex procedures such as total arch replacement or aortic root repair for Stanford type A dissection via a single inverted-T upper mini-sternotomy, beginning in 2016. We also retrospectively compared the outcome of this surgery with that of the same surgery performed through a full sternotomy using a statistical propensity score-matching analysis. The early results of postoperative renal failure and cerebral complications showed no significant differences between the mini-sternotomy and full sternotomy groups, but the respiratory function recovery was faster in the mini-sternotomy group. The CPB time was not prolonged, confirming that upper mini-sternotomy can provide good exposure and allow for adequate manipulation despite the small incision. Moreover, as the surgeon was getting more familiar with this mini access surgery, CPB time has been decreased and it was even shorter than that of full sternotomy group after off-pump reconstruction of LSCA was applied. The cross-clamping time was longer in the mini-sternotomy group than in the full sternotomy group. This might be explained by the fact that aortic root procedures in the upper mini-sternotomy group involved the David procedure using reimplantation technique (4 cases) and Florida sleeve aortic root repair (1 case), which may have taken more time than the Bentall procedure performed in the control group. The ICU and hospital stay were similar between the two groups, as reported in other papers, confirming that the mini-sternotomy approach has an advantage over full sternotomy with respect to recovery.

The mini-sternotomy group showed a low incidence of re-exploration but a 39% higher rate of subxiphoid drainage for late pericardial tamponade. We believe that this was related to inadequate drainage in the early postoperative period because the position of the catheters may have been much higher than in full sternotomy, although we used the same drainage catheter and the same removal protocol in the two groups.

We emphasize that preoperative imaging studies are essential to examine the morphological parameters of the total aortic pathology, especially the level of the aortic root, which can guide how far we do the transverse of sternum. Lentini et al. [14] considered that the diameter of the distal ascending aorta guides the need for circulatory arrest, which they believe argues against a minimally invasive approach. In our experience, however, deep HCA can be performed accessibly and safely (17 of 20 cases), allowing for total arch surgery or frozen elephant trunk implantation for descending aorta reconstruction via an upper mini-sternotomy. We believe that implementing an “arch-first” strategy and using artificial blood vessels with stents would allow easy and safe manipulation of the aortic arch through a small incision. However, the soft tissue incision in the upper portion can be extended toward the neck vessels, allowing greater exposure of the aortic arch, as described in other reports.

**Fig. 2:**
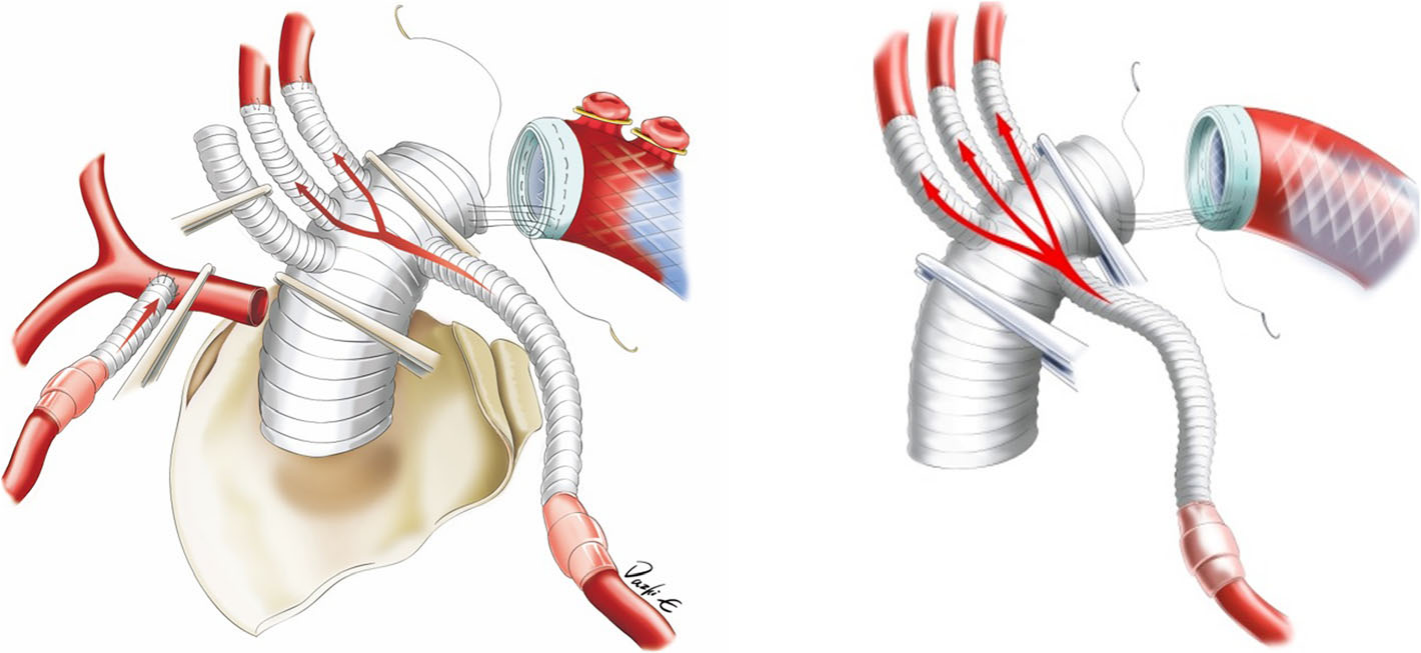
arch first reconstruction and complete antegrade cerebral perfusion during lower body HCA

The arch-first technique in total arch replacement means that anastomoses of the LSCA 、LCA and IA are performed before HCA of the lower body. The LSCA and LCA are anastomosed separately with a four-branch prosthetic graft. Thus, cerebral protection during HCA is achieved by complete antegrade perfusion (Fig. 2). During HCA, a stented graft is implanted into the descending aorta immediately distal to the LSCA. The deep exposure and anastomosis of the descending aorta for reconstruction of the lower body perfusion is the main difficulty for surgeons performing minimally invasive procedures, even through full sternotomy. A stented graft makes this process much easier and safer because the stented graft can be conveniently inserted into the descending aorta. Additionally, the sewing cuff can be extended closer to the median line for better exposure and easier anastomosis to reconstruct the aortic arch, even through an upper mini-sternotomy. In the present study, we used stented grafts for all patients undergoing total arch replacement, whether by mini-sternotomy or full sternotomy.

Learning curve is absolutely essential. The upper mini-sternotomy approach was used for isolated aortic valve replacement in our center beginning in 2009. We gradually extended this technique to patients undergoing ascending aorta replacement, hemi-arch replacement, and then total arch replacement and/or aortic root repair even for acute Stanford A dissection. This approach is being performed for increasingly more patients in our center.

The good results, absence of major complications, shorter ventilation period, and shorter ICU and hospital stay in the present study indicate that the upper mini-sternotomy technique is a feasible and safe approach for ascending aorta surgery with complex procedures for aortic dissection, including total arch replacement and aortic root repair. No patient in our study required conversion from the mini-sternotomy approach to full sternotomy.

The main limitation of the present study is its retrospective and descriptive nature. Additionally, the sample size was not large because we performed a matching analysis. Moreover, we only had early results for the mini-sternotomy technique, and a follow-up comparison should be performed in the future. A randomized study may draw meaningful conclusions about the advantages of the upper mini-sternotomy in this group of patients.

## Conclusions

On the basis of the results of our standard propensity score-matching analysis, we believe that the upper mini-sternotomy approach is safe and beneficial in ascending aorta surgery with complex procedures for aortic dissection, including total arch replacement and aortic root repair after stepwise learning curve. We consider that our antecedent experience will be useful to other surgical teams who explore other minimally invasive techniques.

## Data Availability

Supporting data are available through the corresponding author on reasonable request.

## Abbreviations

BMI: Body mass index
SD: Standard deviation
COPD: Chronic obstructive pulmonary disease
PAD: Peripheral artery disease
CAD: Coronary artery disease
AI: Aortic insufficiency
LVEF: Left ventricular ejection fraction
HTK: Histidine tryptophan ketoglutarate
IA: Innominate artery
LSCA: Left subclavian artery
LCA: Left carotid artery
CPB: Cardiopulmonary bypass
SPSS: Statistical package for social sciences
HCA: Hypothermic circulatory arrest
ICU: Intensive care unit
ASA: Ascending aorta
RBC: Red blood cell

## References

[1] Tabata M, Khalpey Z, Aranki SF (2007). Minimal access surgery of ascending and proximal arch of the aorta: a 9-year experience. Ann Thorac Surg.

[2] Brown ML, McKellar SH, Sundt TM (2009). Ministernotomy versus conventional sternotomy for aortic valve replacement: a systematic review and meta-analysis. J Thorac Cardiovasc Surg.

[3] Gilmanov D, Solinas M, Farneti PA (2015). Minimally invasive aortic valve replacement: 12-year single center experience. Ann Cardiothorac Surg.

[4] Shehada SE, Öztürk Ö, Wottke M, et al. Propensity score analysis of outcomes following minimal access versus conventional aortic valve replacement. Eur J Cardiothorac Surg. 2016;49(2):464–9; discussion 469–70. 10.1093/ejcts/ezv061 25732967

[5] Roselli EE (2015). Interventions on the aortic valve and proximal thoracic aorta through a minimally invasive approach. Ann Cardiothorac Surg.

[6] Tokuda Y, Oshima H, Narita Y (2016). Extended total arch replacement via the L-incision approach: single-stage repair for extensive aneurysms of the aortic arch. Interact Cardiovasc Thorac Surg.

[7] Oishi Y, Sonoda H, Tanoue Y (2011). Advantages of the L-incision approach comprising a combination of left anterior thoracotomy and upper half-median sternotomy for aortic arch aneurysms. Interact Cardiovasc Thorac Surg.

[8] Morisaki A, Hattori K, Kato Y (2015). Evaluation of aortic valve replacement via the right parasternal approach without rib removal. Ann Thorac Cardiovasc Surg.

[9] Filip G, Bryndza MA, Konstanty-Kalandyk J (2018). Ministernotomy or sternotomy in isolated aortic valve replacement? Early results. Kardiochir Torakochirurgia Pol.

[10] Perrotta S, Lentini S (2009). Ministernotomy approach for surgery of the aortic root and ascending aorta. Interact Cardiovasc Thorac Surg.

[11] Bonacchi M, Prifti E, Giunti G (2002). Does ministernotomy improve postoperative outcome in aortic valve operation? A prospective randomized study. Ann Thorac Surg.

[12] Svensson LG, Nadolny EM, Kimmel WA (2001). Minimal access aortic surgery including re-operations. Eur J Cardiothorac Surg.

[13] Goebel N, Bonte D, Salehi-Gilani S (2017). Minimally Invasive Access Aortic Arch Surgery. Innovations (Phila).

[14] Lentini S, Specchia L, Nicolardi S (2016). Surgery of the Ascending Aorta with or without Combined Procedures through an Upper Ministernotomy: Outcomes of a Series of More Than 100 Patients. Ann Thorac Cardiovasc Surg.

